# Supplementary material for: GORAB scaffolds COPI at the trans-Golgi for efficient enzyme recycling and correct protein glycosylation
Source: Nat Commun. 2019 Jan 10;10:127. doi: 10.1038/s41467-018-08044-6 (PMC6328613; doi:10.1038/s41467-018-08044-6)
Supplement: Supplementary file 1 — Supplementary Information [file 41467_2018_8044_MOESM1_ESM.pdf]

Supplementary Information for

GORAB scaffolds COPI at the *trans*-Golgi for efficient enzyme recycling and correct protein glycosylation

Witkos et al

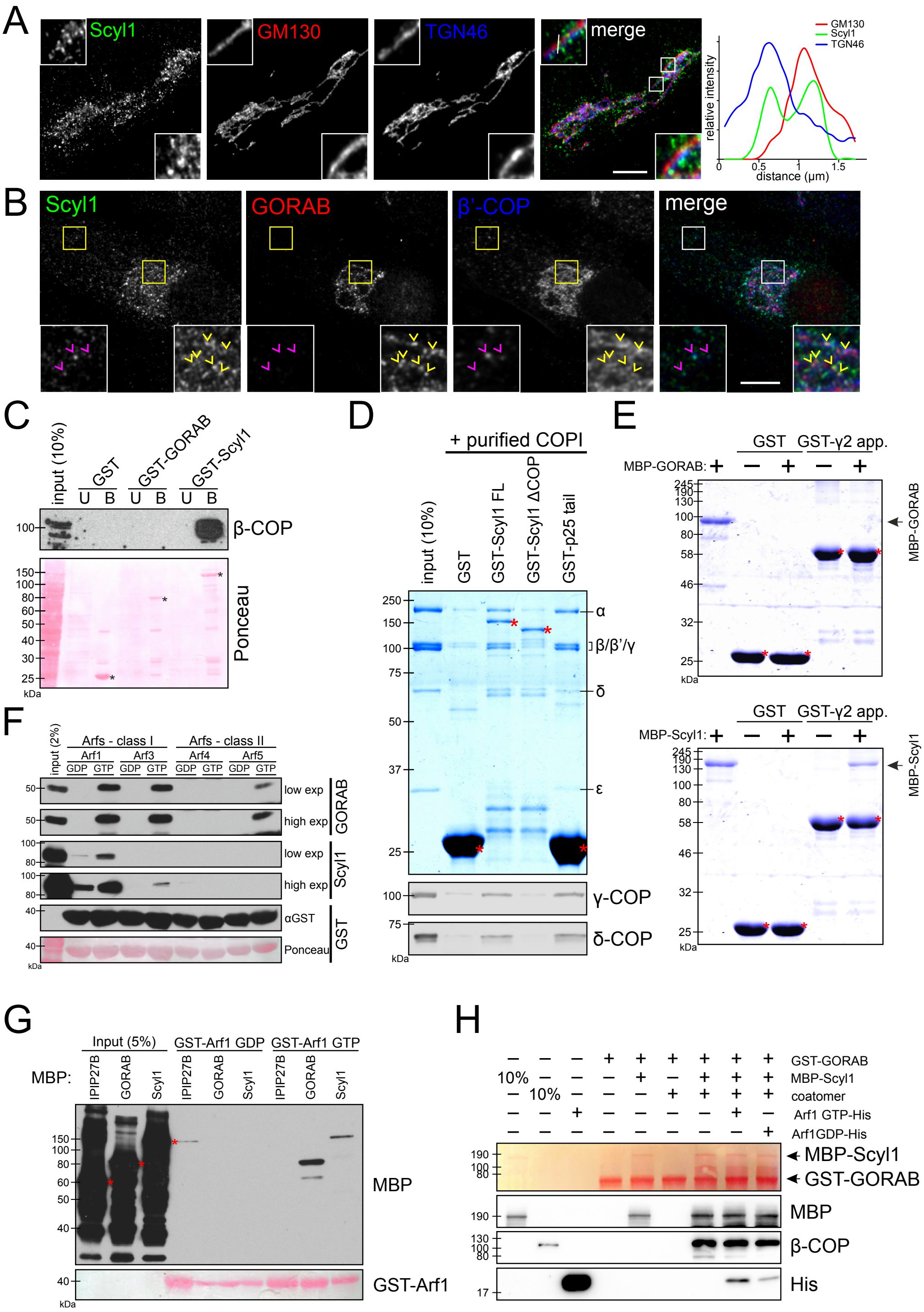

Supplementary Figure 1. Scyl1 localization and GORAB/Scyl1 binding to coatomer and Arf. A. Scyl1 localization in the Golgi. Human dermal fibroblasts were fixed and labeled with antibodies against Scyl1, GM130 and TGN46. Scale bar, 10 μm. The white line indicates the pixels used for the RGB fluorescence intensity profile plot depicted on the right, and is representative of n=20 cells. B. Co-localization analysis of GORAB, Scyl1 and β'-COP. Human dermal fibroblasts were fixed and labeled with antibodies against Scyl1, GORAB and β'-COP. Scale bar, 10 μm. Yellow arrowheads mark GORAB puncta co-localizing both with Scyl1 and β'-COP, magenta arrowheads mark GORAB puncta co-localizing with Scyl1 only. C. Pull-down using purified GST, GST-GORAB, GST-Scyl1 as bait and sHeLa lysate. Input (I, 5%), unbound (U, 50%), and bound fractions (B, 50%) were blotted for the β-COP subunit of coatomer. GST-tagged proteins were visualized by staining with Ponceau S and bands representing GST-tagged bait proteins are marked with asterisks. D. Pull-down using purified GST, GST-Scyl1 FL, GST-Scyl1ΔCOP and GST-p25 tail as bait and purified recombinant coatomer as prey. Samples were subjected to SDS-PAGE and stained with Coomassie Blue or blotted for γ-COP and δ-COP. GST-tagged bait proteins are marked with asterisks. E. In vitro binding assay between purified GST and GST-γ-2 appendage domain, and MBP-GORAB and MBP-Scyl1. Samples were subjected to SDS-PAGE and stained with Coomassie Blue. GST-tagged bait proteins are marked with asterisks. F. Pull-down using purified GST-Δ14Arf1 (GDP-T31N; GTP-Q71L), GST-Δ14Arf4 (GDP-T31N; GTP-Q71L) and sHeLa cell lysate. Samples, input (2%) and bound fractions (50%), were blotted for GORAB, Scyl1 and GST. G. An in vitro binding using purified GST-Δ14Arf1 and MBP-IPI27B, MBP-GORAB and MBP-Scyl1. Samples, input (2%), bound fractions (50%), were blotted for MBP and GST-Arf1 was visualized by staining with Ponceau S. Full-length MBP-tagged proteins in the inputs are marked with asterisks. H. In vitro binding assay between purified GST-GORAB, MBP-Scyl1, recombinant coatomer and Arf1 (GDP-T31N; GTP-Q71L)-His. Samples were blotted for MBP, β-COP and His. GST-GORAB and MBP-Scyl1 were visualized by staining with Ponceau S.

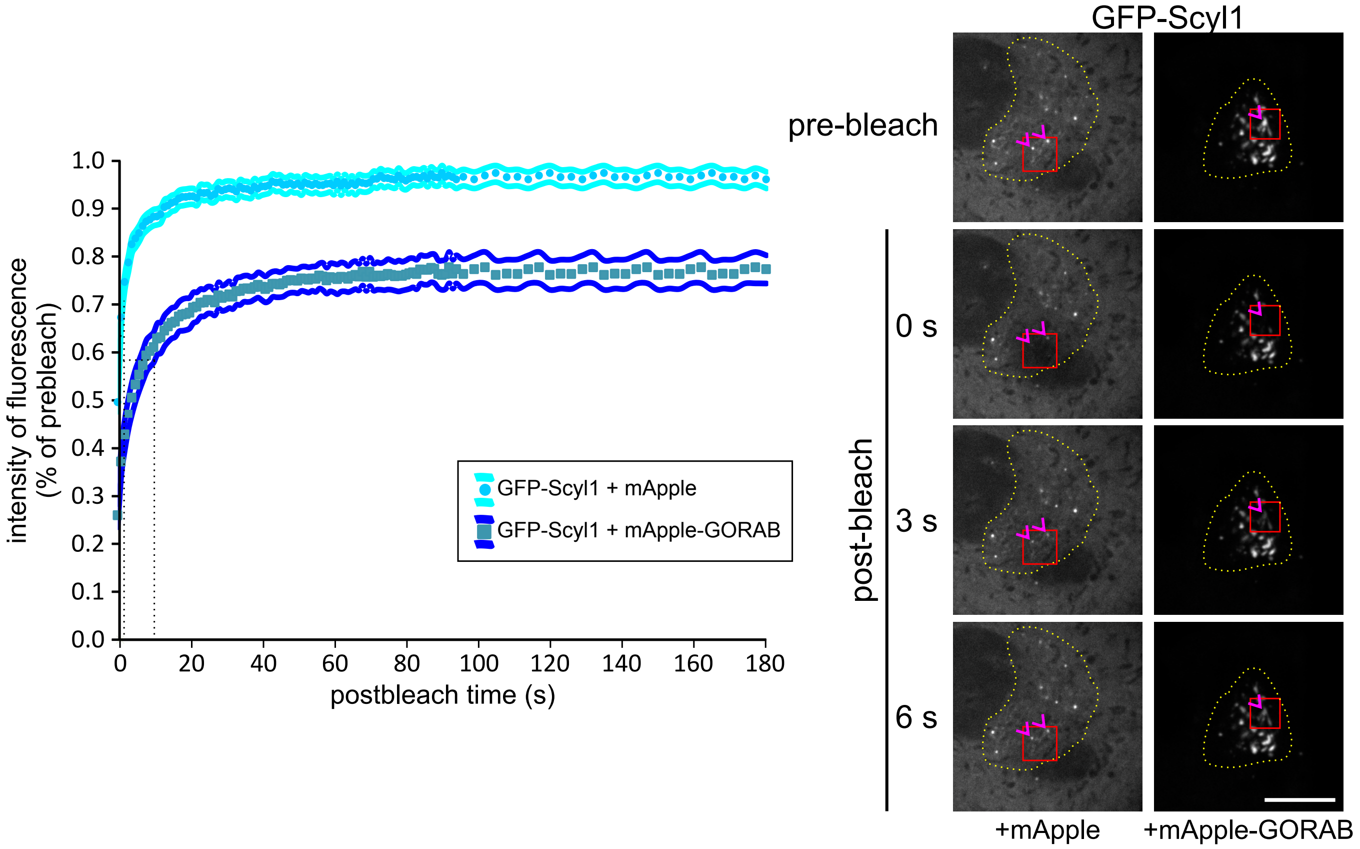

Supplementary Figure 2. FRAP analysis of Scyl1 dynamics. Fluorescence recovery after photobleaching. Left, FRAP recovery curves for GFP-Scyl1 in cells co-expressing mApple or mApple-GORAB. Means with SEM for GFP-Scyl1 co-expressed with mApple (n = 43 cells) and GFP-Scyl1 co-expressed with mApple-GORAB (n = 37 cells). Dotted lines mark points of half-time recoveries. Right, representative HeLaM GFP-Scyl1 co-expressing mApple or mApple-GORAB cells at pre-bleached and selected post-bleached states. Dotted yellow line marks the Golgi area, bleached region of interests are marked with red boxes and GFP-Scyl1 structures are labeled with magenta arrowheads. Scale bar, 10 μm.

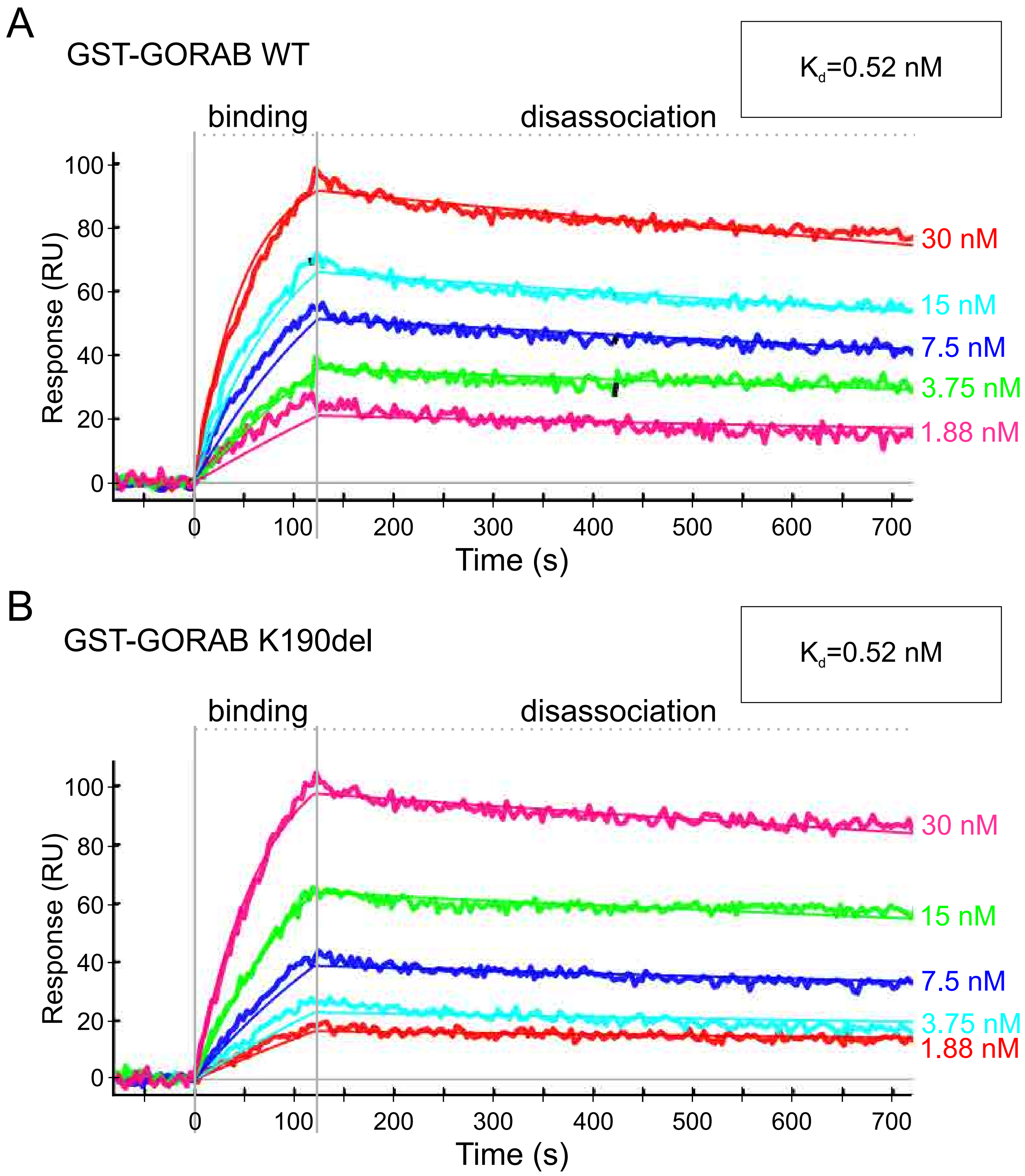

Supplementary Figure 3. SPR measurement of GORAB-Scyl1 binding. Binding of GST-GORAB WT (A) and K190del (B) variants to MBP-Scyl1 attached to the GLH sensor chip via cross-linked anti-MBP antibody using surface plasma resonance. Binding took place for 120 s followed by 600 s dissociation phase. Concentrations of analyte (GST-tagged GORAB) during the binding phase are indicated on the right. Similar results were obtained in three separate experiments, which allowed determination of the Kd (top right corner).

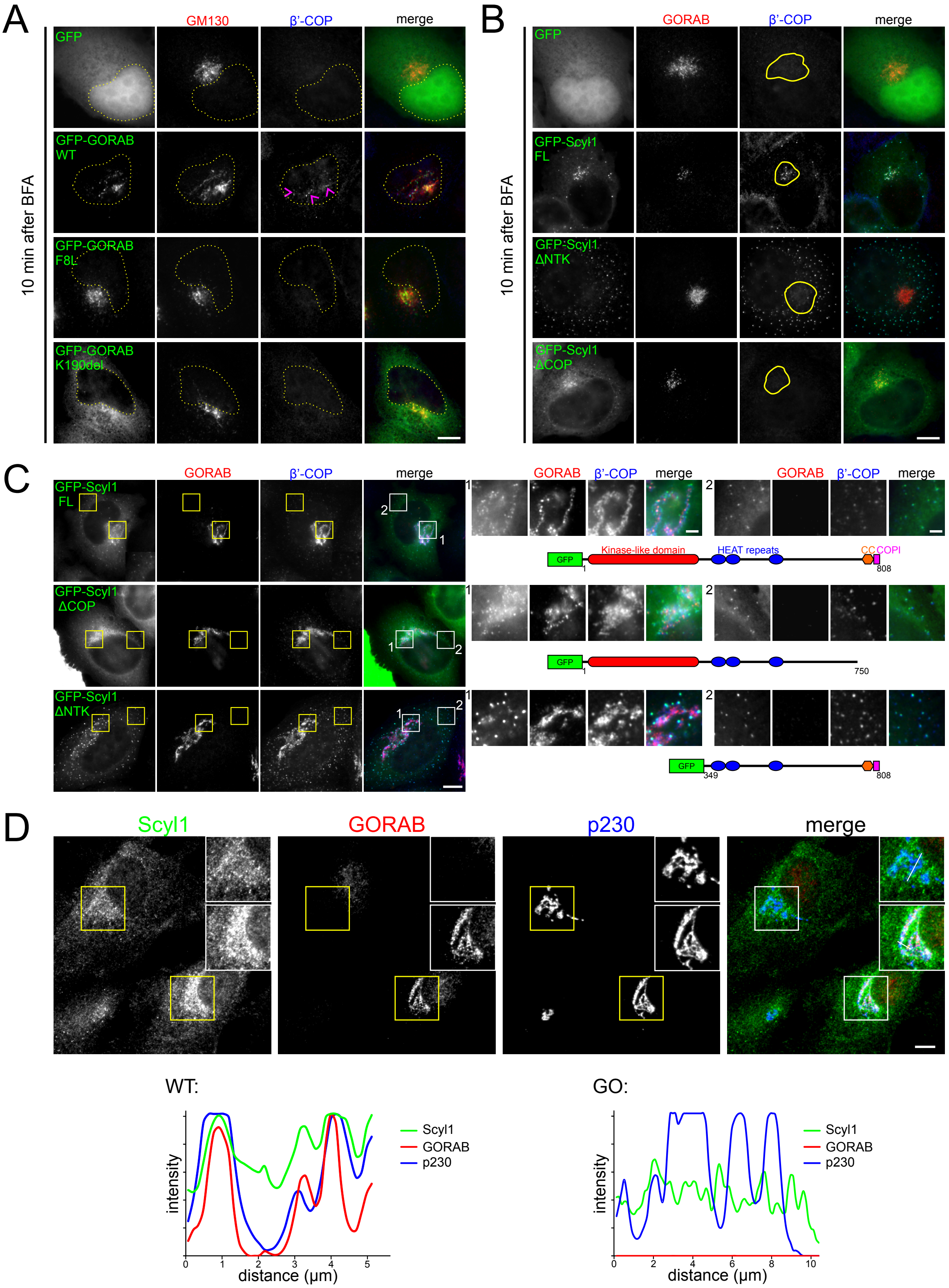

Supplementary Figure 4. Stabilization of coat protein binding at the Golgi and ERGIC by GORAB and Scyl1 variants. A. Effects of GFP-GORAB variants on Golgi retention of COPI in BFA-treated cells. HeLaM cells were transiently transfected with GFP or GFP-GORAB variants and exposed to 5 µg/mL BFA for 10 min prior to fixation. Cells were labeled with antibodies to GM130 and β'-COP. Scale bar, 10 µm. Dotted line marks the position of the nucleus and β'-COP positive structures in the Golgi are marked with magenta arrowheads. B. Effects of GFP-Scyl1 truncation constructs on Golgi retention of COPI in BFA-treated cells. HeLaM cells were transiently transfected with GFP or GFP-Scyl1 truncation constructs and exposed to 5 µg/mL BFA for 10 min prior to fixation. Cells were labeled with antibodies to GM130 and β'-COP. Scale bar, 10 µm. The peri-Golgi area is marked by a solid yellow line. C. Subcellular localization of GFP-tagged Scyl1 truncated constructs in untreated cells. HeLaM cells transiently expressing GFP-tagged Scyl1 truncation constructs were fixed and labeled with antibodies to GORAB and β'-COP. Scale bar, 10 µm. Insets show magnifications of the regions boxed on the left and schematic depictions of the corresponding deletion constructs. . Scale bar, 2 µm. D. Subcellular localization of Scyl1 in WT and GO fibroblasts. Cells were fixed and labeled with antibodies to GORAB and p230. Scale bar, 10 µm. The white lines in the insets indicate the pixels used for the RGB fluorescence intensity profile plots shown underneath, and are representative of n=20 cells.

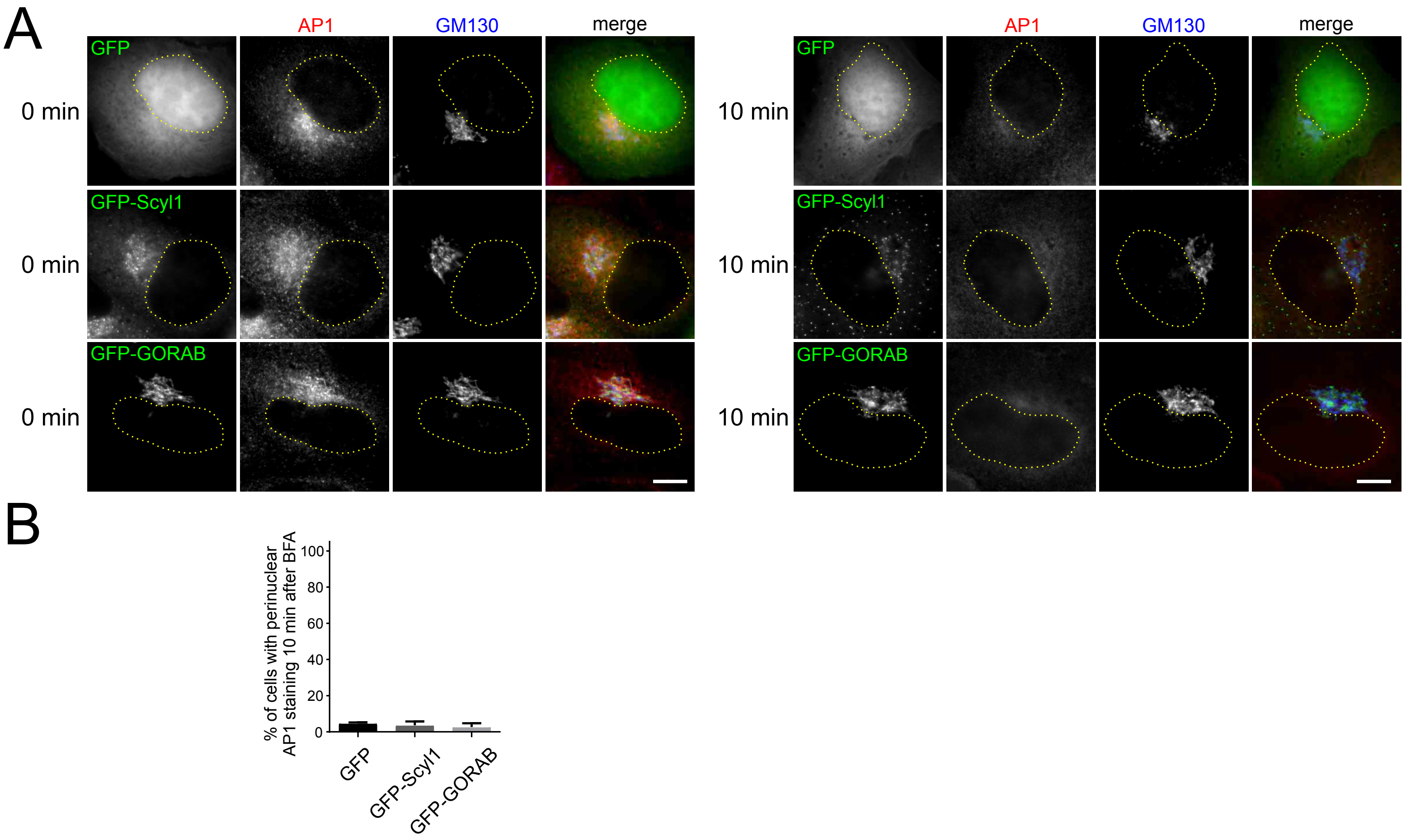

Supplementary Figure 5. AP1 membrane association is not affected by GORAB or Scyl1. A. AP1 subcellular localization in HeLaM cells transfected with GFP, GFP-GORAB or GFP-Scyl1 constructs and treated with 5 µg/mL BFA for 10 min. Cells were labeled with antibodies to AP1 and GM130. Scale bar, 10 µm. B. Quantification of HeLaM cells with AP1 retention in the peri-Golgi area following, 10 min incubation with 5 µg/mL BFA. Error bars represent mean ± SD, n=100 cells in each of 3 independent experiments, unpaired t-test.

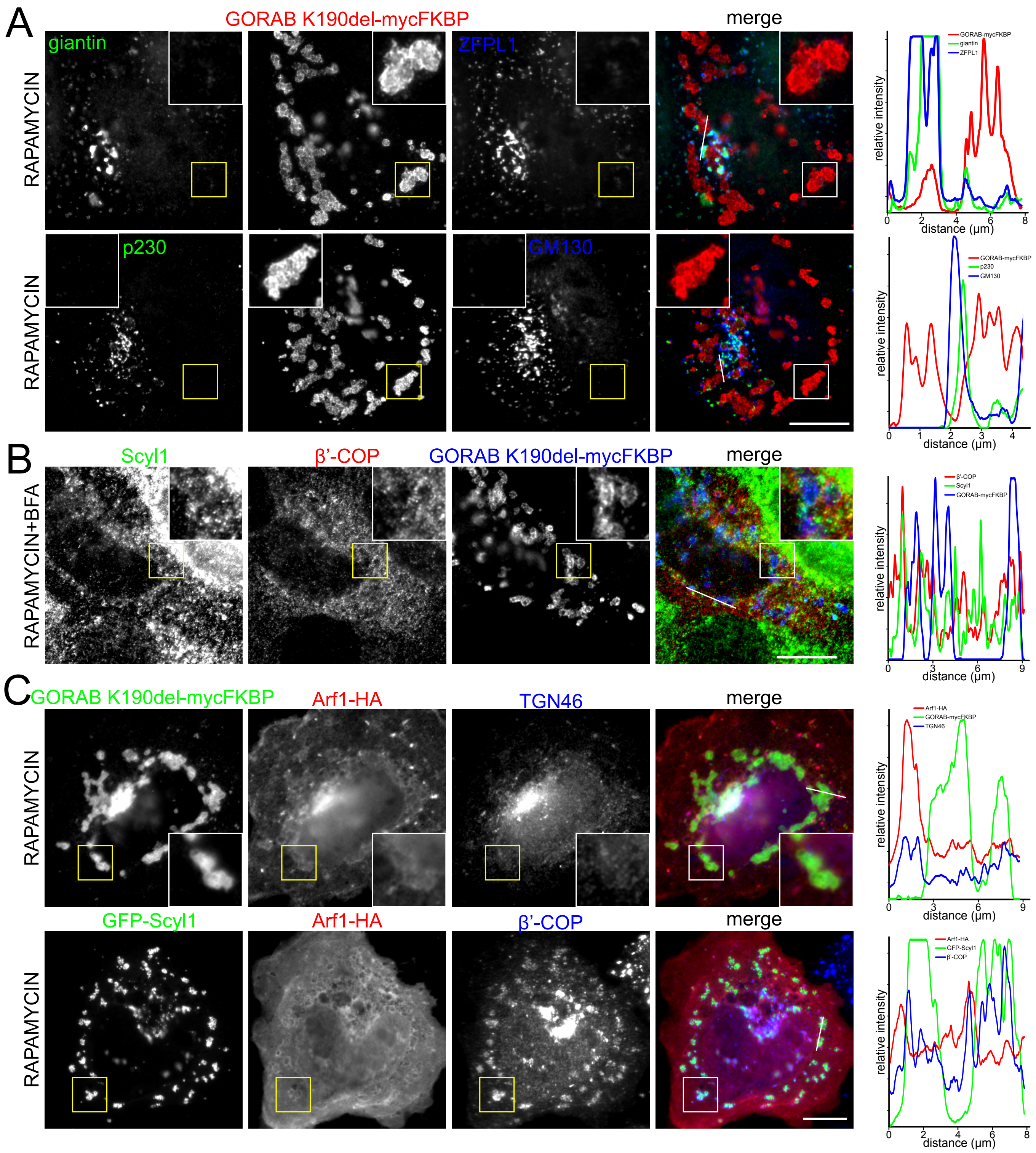

Supplementary Figure 6. Mitochondrial relocation assay for Golgi markers, Scyl1 and Arf1. A. HeLaM cells co-transfected with mito-FRB and GORABK190del-mycFKBP constructs were pretreated with 2.5 μg/mL nocodazole for 2 h and further incubated with 1 μM rapamycin or DMSO for 3 h prior to fixation. Cells were labeled with antibodies to the Golgi markers giantin, ZFPL1, p230 and GM130. B. HeLaM cells co-transfected with mito-FRB and GORABK190del-mycFKBP and treated as described in A were labeled with antibodies to myc and endogenous Scyl1 and β'-COP. C. HeLaM cells co-transfected with mito-FRB and GORABK190del-mycFKBP and treated as described in A were labeled with antibodies to myc, HA and the Golgi marker TGN46 (top row) or β'-COP (bottom row). Scale bars, 10 μm. The white lines indicate pixels used for the RGB fluorescence intensity profile plots shown on the right, and are representative of n=20 cells.

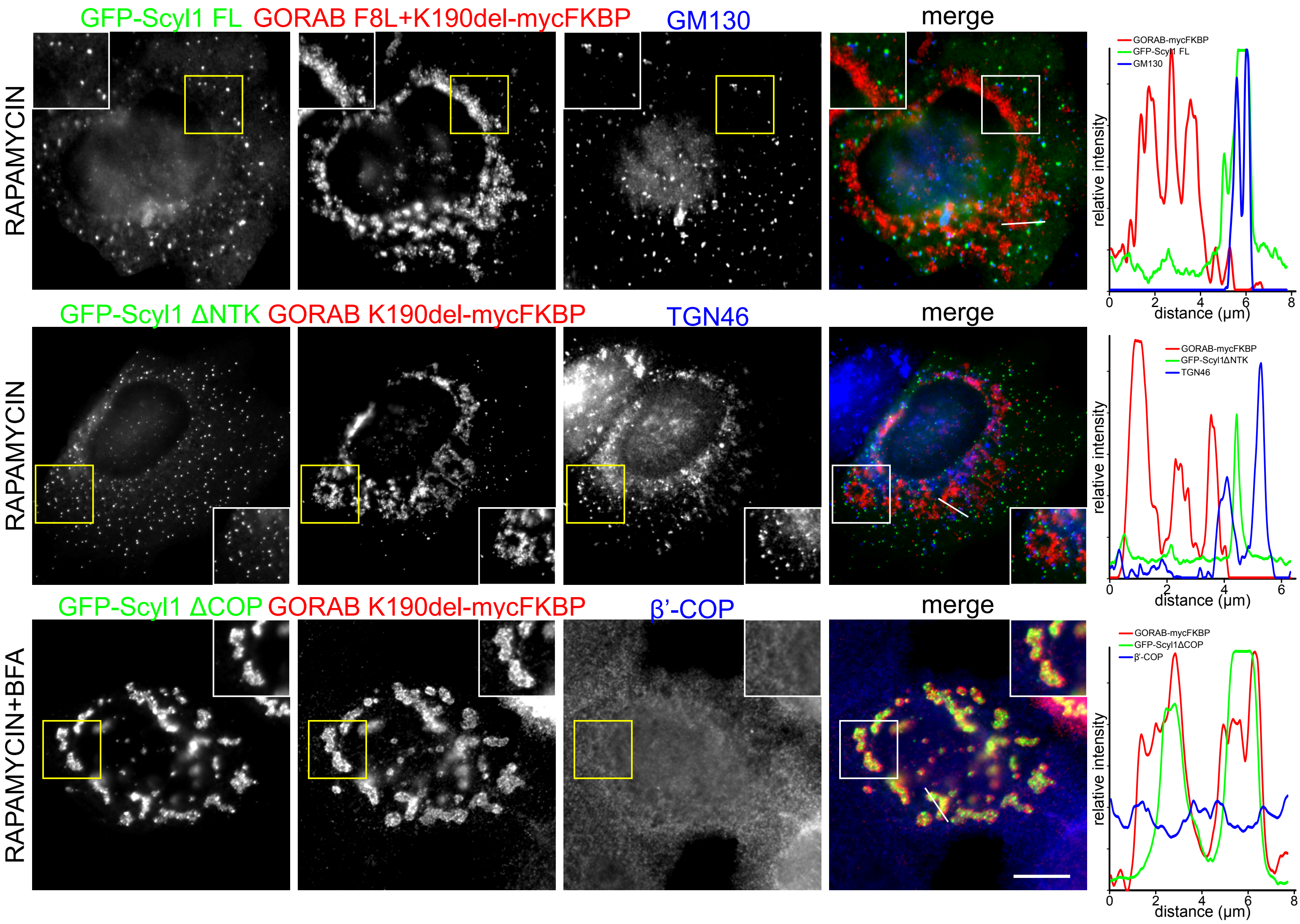

Supplementary Figure 7. Mitochondrial relocation of COPI requires Scyl1 binding to GORAB and COPI. HeLaM cells co-transfected with mito-FRB, GORABK190del-mycFKBP or GORABF8L+K190del-mycFKBP and GFP-Scyl1 constructs were pretreated with 2.5 µg/mL nocodazole for 2 h and further incubated with 1 µM rapamycin for 3 h without (upper 2 rows) or with incubation with 5 µg/mL BFA for 15 min (bottom row) prior to fixation. Cells were labeled with antibodies to myc, GM130, TGN46 or β'-COP as indicated. Scale bar, 10 µm. The white line indicates the pixels used for the RGB fluorescence intensity profile plots depicted on the right

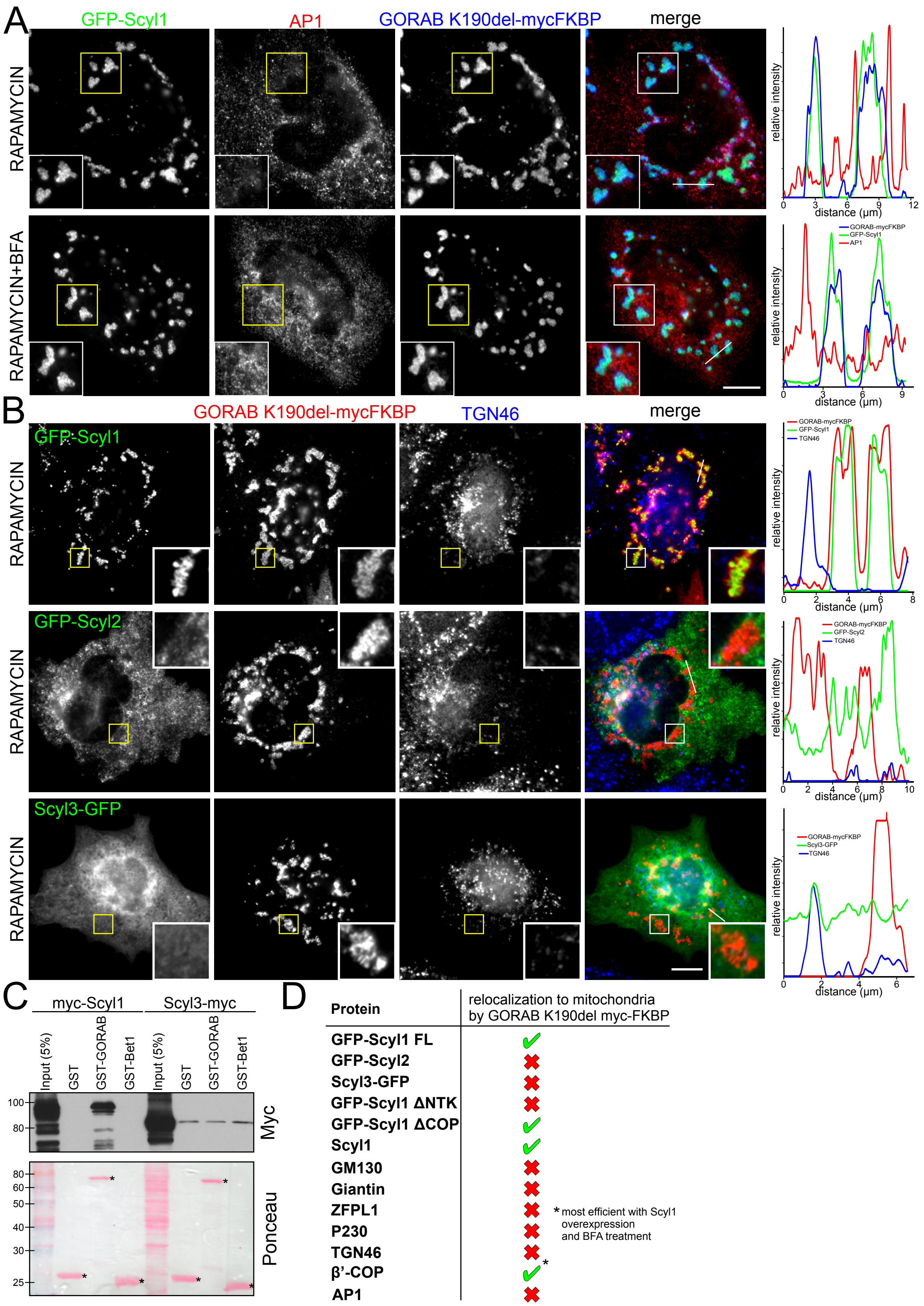

Supplementary Figure 8. Mitochondrial relocation assay for AP1 and Scyl1. HeLaM cells co-transfected with mito-FRB, GORABK190del-mycFKBP and GFP-Scyl1 constructs were pretreated with 2.5 µg/mL nocodazole for 2 h and further incubated with 1 µM rapamycin for 3 h without (top row) or with incubation with 5 µg/mL BFA for 15 min (bottom row) prior to fixation. Cells were labeled with antibodies to AP1 and myc. B. HelaM cells were co-transfected with mito-FRB, GORABK190del-mycFKBP and GFP-Scyl1, GFP-Scyl2 or Scyl3-GFP, treated as described in A and labeled with antibodies to myc and the Golgi marker TGN46. In panels A-B, scale bars are 10 µm and white lines indicate the pixels used for the RGB fluorescence intensity profile plots shown on the right, and are representative of n=20 cells. C. Pull-down using purified GST, GST-GORAB or GST-Bet1 as bait and lysate from HeLaM cells transiently expressing myc-Scyl1 and Scyl3-myc. Samples were subjected to SDS-PAGE and stained with Coomassie Blue or blotted for myc. GST-tagged bait proteins are marked with asterisks. D. A table summarizing mitochondrial recruitment experiments with GORABK190del-mycKFBP.

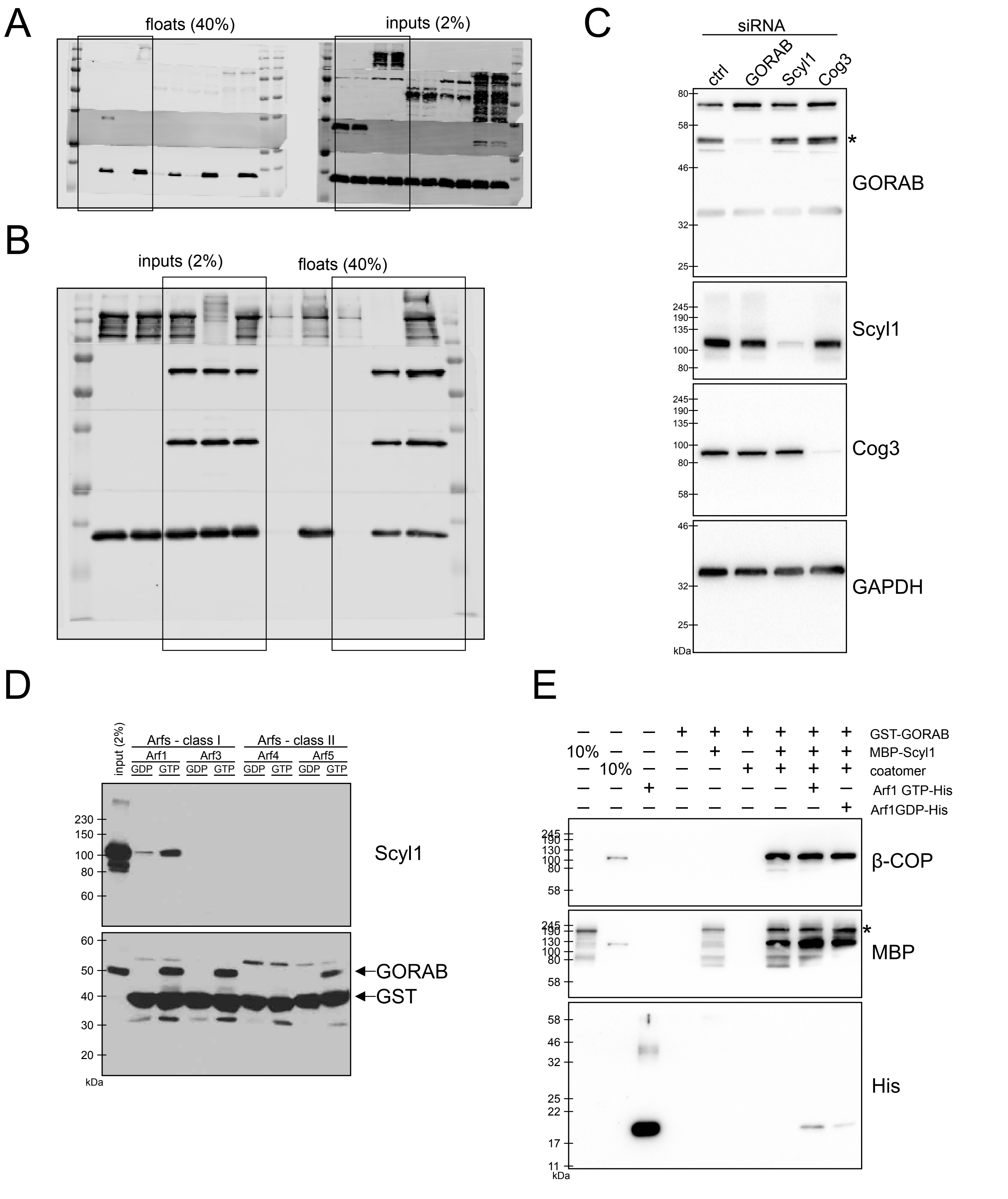

Supplementary Figure 9. Uncropped blots. A. Raw data from Fig 6A. B. Raw data from Fig 6B C. Raw data from Fig 8A. D. Raw data from Fig S1F. E. Raw data from Fig S1H.

Supplementary Table 1. Assignment of N-glycan structures in the N-glycome analysis. Shown are the glycan assignments for control and *Gorab*^{Null} mouse skin tissue (Figure 7G).

**Calculated (*m/z*)**	**Measured (m/z)**	**Mass accuracies (ppm)**	**Assignment**	**Type**[1]
1141,5724	1141.5704	1.7	$Fuc_1Hex_2HexNAc_2 + Na^+$	DP
1171,5830	1171.5840	0.8	$Hex_3HexNAc_2 + Na^+$	DP
1345,6722	1345.6737	1.1	$Fuc_1Hex_3HexNAc_2 + Na^+$	DP
1375,6828	1375.6807	1,5	$Hex_4HexNAc_2 + Na^+$	DP
1416,7093	1416.7048	3,1	$Hex_3HexNAc_3 + Na^+$	C
1579.7826	1579.7861	2,2	$Hex_5HexNAc_2 + Na^+$	O
1620,8091	1620.8146	3,4	$Hex_4HexNAc_3 + Na^+$	C
1661,8357	1661.8384	1,6	$Hex_3HexNAc_4 + Na^+$	C
1783,8823	1783.8852	1,6	$Hex_6HexNAc_2 + Na^+$	O
1835,9249	1835.9289	2,1	$Fuc_1Hex_3HexNAc_4 + Na^+$	C
1865,9354	1865.9395	2,2	$Hex_4HexNAc_4 + Na^+$	C
1906,9620	1906.9666	2,4	$Hex_3HexNAc_5 + Na^+$	C
1987,9821	1987.9776	2,2	$Hex_7HexNAc_2 + Na^+$	O
2040,0246	2040.0326	3,9	$Fuc_1Hex_4HexNAc_4 + Na^+$	C
2070,0352	2070,0469	5,6	$Hex_5HexNAc_4 + Na^+$	C
2192,0819	2192.0938	5,4	$Hex_8HexNAc_2 + Na^+$	O
2244,1244	2244.1295	2,2	$Fuc_1Hex_5HexNAc_4 + Na^+$	C
2285,1510	2285.1567	2,5	$Fuc_1Hex_4HexNAc_5 + Na^+$	C
2396,1816	2396,1765	2,1	$Hex_9HexNAc_2 + Na^+$	O
2431,2089	2431,2148	2,4	$NeuAc_1Hex_5HexNAc_4 + Na^+$	C
2448,2242	2448,2183	2,4	$Fuc_1Hex_6HexNAc_4 + Na^+$	C
2489,2507	2489.2385	4,9	$Fuc_1Hex_5HexNAc_5 + Na^+$	C
2605,2981	2605,3016	1,3	$NeuAc_1Fuc_1Hex_5HexNAc_4 + Na^+$	C
2635,3087	2635,3043	1,6	$NeuGc_1Fuc_1Hex_5HexNAc_4 + Na^+$	C
2652,3240	2652,3116	4,6	$Fuc_1Hex_7HexNAc_4 + Na^+$	C
2792,3825	2792,3757	2,4	$NeuAc_2Hex_5HexNAc_4 + Na^+$	C
2809,3978	2809,3842	4,8	$NeuAc_1Fuc_1Hex_6HexNAc_4 + Na^+$	C
2822,3840	2822,3901	2,1	$NeuAc_1NeuGc_1Hex_5HexNAc_4 + Na^+$	C
2852,4037	2852.4189	5,3	$NeuGc_2Hex_5HexNAc_4 + Na^+$	C
2966,4717	2966,4785	2,3	$NeuAc_2Fuc_1Hex_5HexNAc_4 + Na^+$	C
2996,4824	2996,4929	3,5	$NeuAc_1NeuGc_1Fuc_1Hex_5HexNAc_4 + Na^+$	C
3026,4929	3026.4997	2,2	$NeuGc_2Fuc_1Hex_5HexNAc_4 + Na^+$	C
3271,6193	3271,6262	2,1	$NeuAc_1NeuGc_1Hex_6HexNAc_5 + Na^+$	C

[1] Glycans were grouped as oligomannose (O), hybrid (H), complex (C) and degradation product (DP - small glycans that are not produced by the mammalian N-glycosylation machinery and therefore most likely originate from lysosomal degradation)

Supplementary Table 2. List of primary antibodies used in this study.

Antigen	Species	Dilution[1]	Name/source of antibody
TGN46	sheep	1:200	Dr. Sreenivasan Ponnambalam, University of Leeds, Leeds, United Kingdom
Scyl1	rabbit	IF: 1:100, WB: 1:500	Dr. Gustav Lienhard, Dartmouth Medical School, NH [1]
P230	mouse	IF 1:50, WB: 1:500	BD Biosciences (611281; Clone 15)
α-tubulin	mouse	1:1 000	Dr. Keith Gull, University of Oxford, Oxford, United Kingdom
GFP	mouse	1:1 000	Thermo Fisher Scientific (MA5-15256; GF28R)
GFP	sheep	1:1 000	[2]
GM130	mouse	1:500	BD Biosciences (610823; Clone 35)
GM130	rabbit	1:500	Anti-N73pep [3]
β-COP	mouse	1:1 000	EAGE [4]
β'-COP	mouse	1:200	CMIA10 [5]
γ1/2-COP	rabbit	1:1 000	γ1R, [6]
δ-COP	rabbit	1:1 000	877 [7]
ε-COP	rabbit	1:1 000	[8]
Arf1	rabbit	1:1 000	C1 [9]
MBP	mouse	1:10 000	New England Biolabs (E8032)
GAPDH	mouse	1:4 000	Santa Cruz (sc-365062; G-9)
GST	sheep	1:10 000	[10]
MtHsp70	mouse	1:400	Thermo Fisher Scientific (MA3-028; JG1)
Myc	mouse	1:1 000	Cell Signalling Technology (2276S; 9B11)
Myc	goat	1:2 000	Abcam (ab9132)
Cog3	mouse	1:1 000	Dr. Vladimir Lupashin, University of Arkansas for Medical Sciences, AR
AP-1	mouse	1:200	Thermo Fisher Scientific (MA1-25066; clone 100/3)
His	rabbit	1:1 000	Abcam (ab9108)
HA	rat	1:1 000	Sigma (11867423001; clone 3F10)
ZFPL1	sheep	1:200	[11]
Giantin	rabbit	1:400	Dr. Manfred Renz, Institute of Immunology and Molecular Genetics, Karlsruhe, Germany
GORAB	rabbit	WB: 1:500	Proteintech (17798-1-AP)
GORAB	rabbit	IF: 1:250	raised against human GORAB by injecting an N-terminal GST-tagged GORAB fragments (amino acids 1-130 and 301-369) into rabbit and sheep, respectively (conducted by the Scottish National Blood Transfusion Service, Edinburgh, United Kingdom)
GORAB	sheep	IF: 1:250	

[1] IF – immunofluorescence, WB – western blotting

Supplementary Table 3. List of primers used in this study.

Name	Sequence
GORABclone_F	TGCGAGATTTGGGCACTT
GORABclone_R	AGGGCATGATCTACTGCA
GORAB131_XhoI_F	CCGCTCGAGTGCAAATTGGAGAAAAAG
GORAB130_NotI_R	ATAAGAATGCGGCCGCTCAATCTGGCTTTGGAGGTAG
GORAB369_NotI_R	ATAAGAATGCGGCCGCTCATGTGGCCAAAGCAGCTGA
GORAB130_HindIII_R	CCCAAGCTTTCAATCTGGCTTTGGAGGTAG
GORAB300_HindIII_R	CCCAAGCTTTCATGATTCTACTTCTTGTTC
GORAB369_HindIII_R	CCCAAGCTTTCATGTGGCCAAAGCAGCTGA
GORAB301_XhoI_F	CCGCTCGAGAGGAGACCAGTGGTTCGT
GORAB_XhoI_F	GATCCTCGAGATGGCGCAAGGTTGG
Scyl1_Y2H_F	GATCCATATGATGTGGTTCTTTGCCCGGGACCC
Scyl1_Y2H_R	GATCGAATTCTCAGTCCAGCTTCCGGGCTCCC
Scyl1_GFP-C1_F	GATCGAATTCTATGTGGTTCTTTGCCCGGGA
Scyl1_GFP-C1_R	GATCGGTACCTCAGTCCAGCTTCCGGGC
Scyl1_NcoI_F	GATCCCATGGATTGGTTCTTTGCCCGGGACCC
Scyl1_348_R	GATCGGTACCTCACTGCTGATACTCCTCAGCGCTC
Scyl1_750R	CAGTGGTACCTCACGGCTGGGTGCTGGGAC
Scyl1_349_F	GATCGAATTCTAAGATCATCCCTGTGGTGGTCAAG
Scyl1_547_F	GATCGAATTCTCTGGAGGAAGTGGAGAAGGATG
Scyl1_MBP_F	GATCGAATTCATGTGGTTCTTTGCCCGGGA
Scyl1_MBP_R	GATCTCTAGATCAGTCCAGCTTCCGGGCT
Arf3_GST_F	GATCGGATCCAAGAAGGAGATGCGCATC
Arf3_GST_R	GATCCTCGAGTCACTTCTTGTTTTTGAGCTGATTG
Arf4_GST_F	GATCGGATCCAAGAAGCAGATGCGCA
Arf4_GST_R	GATCCTCGAGTTAACGTTTTGAAAGCTCATTTGAC
Arf5_GST_F	GATCGGATCCAAGAAGCAGATGCGGA
Arf5_GST_R	GATCCTCGAGTTAGCGCTTTGACAGCTC
Arf1_His_F	GATCGAATTCATGGGGAACATCTTCGCC
Arf1_His_R	GATCCTCGAGCTTCTGGTTCCGGAGCTG
Arf1_Q71L_mut_F	GGACGTGGGTGGCTTGGACAAGATCCGGCC
Arf1_Q71L_mut_R	GGCCGGATCTTGTCCAAGCCACCCACGTCC
Arf1_T31N_mut_F	GGATGCTGCAGGGAAGAACACGATCCTCTACAAGCTTAAGC
Arf1_T31N_mut_R	GCTTAAGCTTGTAGAGGATCGTGTTCTTCCCTGCAGCATCC
GORAB_F8Lmut_F	CAAGGTTGGGCAGGCTTGTCTGAGGAGGAACTGAG
GORAB_F8Lmut_R	CTCAGTTCCTCCTCAGACAAGCCTGCCCAACCTTG
GORAB_K190delmut_F	CCATGAAACTAAAGCGGATCCAGGAGTTGCAGGCTTTAGATG
GORAB_K190delmut_R	CATCTAAAGCCTGCAACTCCTGGATCCGCTTTAGTTTCATGG
Arf1_HA_F	GATCGGATCCATGGGGAACATCTTCGCCAACCTC
Arf1_HA_R	GATCCCGCGGCCGCCTTCTGGTTCCGGAGCTGATTGGACAGC
Scyl3_GFP_F	GATCCTCGAGAGATCTGCTTGGCTTTGAG
Scyl3_GFP_R	GATCGGATCCCCAGTTATTATCTTCCCAG

Supplementary references

1 Liu, S. C., Lane, W. S. & Lienhard, G. E. Cloning and preliminary characterization of a 105 kDa protein with an N-terminal kinase-like domain. *Biochim Biophys Acta* **1517**, 148-152 (2000).

2 Diao, A., Rahman, D., Pappin, D. J., Lucocq, J. & Lowe, M. The coiled-coil membrane protein golgin-84 is a novel rab effector required for Golgi ribbon formation. *J Cell Biol* **160**, 201-212, doi:10.1083/jcb.200207045 (2003).

3 Nakamura, N., Lowe, M., Levine, T. P., Rabouille, C. & Warren, G. The vesicle docking protein p115 binds GM130, a cis-Golgi matrix protein, in a mitotically regulated manner. *Cell* **89**, 445-455 (1997).

4 Pepperkok, R. *et al.* Beta-COP is essential for biosynthetic membrane transport from the endoplasmic reticulum to the Golgi complex in vivo. *Cell* **74**, 71-82 (1993).

5 Palmer, D. J., Helms, J. B., Beckers, C. J., Orci, L. & Rothman, J. E. Binding of coatomer to Golgi membranes requires ADP-ribosylation factor. *J Biol Chem* **268**, 12083-12089 (1993).

6 Pavel, J., Harter, C. & Wieland, F. T. Reversible dissociation of coatomer: functional characterization of a beta/delta-coat protein subcomplex. *Proc Natl Acad Sci U S A* **95**, 2140-2145 (1998).

7 Faulstich, D. *et al.* Architecture of coatomer: molecular characterization of delta-COP and protein interactions within the complex. *J Cell Biol* **135**, 53-61 (1996).

8 Hara-Kuge, S. *et al.* En bloc incorporation of coatomer subunits during the assembly of COP-coated vesicles. *J Cell Biol* **124**, 883-892 (1994).

9 Reinhard, C., Schweikert, M., Wieland, F. T. & Nickel, W. Functional reconstitution of COPI coat assembly and disassembly using chemically defined components. *Proc Natl Acad Sci U S A* **100**, 8253-8257, doi:10.1073/pnas.1432391100 (2003).

10 Hyvola, N. *et al.* Membrane targeting and activation of the Lowe syndrome protein OCRL1 by rab GTPases. *EMBO J* **25**, 3750-3761, doi:10.1038/sj.emboj.7601274 (2006).

11 Chiu, C. F. *et al.* ZFPL1, a novel ring finger protein required for cis-Golgi integrity and efficient ER-to-Golgi transport. *EMBO J* **27**, 934-947, doi:10.1038/emboj.2008.40 (2008).
